# A perspective of scale differences for studying the green total factor productivity of Chinese laying hens

**DOI:** 10.1038/s41598-022-10693-z

**Published:** 2022-04-27

**Authors:** Shen Zhong, Xi Chen, Junwei Li, Shubo Jiang

**Affiliations:** 1 School of Finance, Harbin University of Commerce, Harbin City, Heilongjiang 150000 People’s Republic of China; 2 School of Economics, Harbin University of Commerce, Harbin City, Heilongjiang 150000 People’s Republic of China

**Keywords:** Ecology, Agroecology, Environmental economics

## Abstract

In people’s daily life, egg is one of the main animal protein foods, which will produce different emissions on its breeding procedure. Therefore, in order to promote the development of China’s layer industry, it is necessary to reduce pollutant emissions by improving efficiency. This paper uses Minimum distance to weak efficient frontier-Metafrontier Malmquist Luenberger (MinDW-MML) index model, by considering environmental factors and scale heterogeneity, to explore the evolution characteristics of laying hens breeding green total factor productivity (LHG) in China based on the data of 24 major laying provinces (municipalities) from 2004 to 2018. The results show that: (1) From 2004 to 2018, medium-scale LHG in China is the highest, the small-scale is the second, and the large-scale is the lowest. In the light of regional distribution, the western region is the highest, followed by the central region, and the eastern region is the lowest. (2) From 2004 to 2018, the overall China’s LHG showed a positive growth, and the decomposition indicators were characterized by decreased efficiency and technological progress. In general, the layer industry is vulnerable and easily affected by external factors. (3) Results from common frontiers and group frontiers exist some differences. The LHG under the common frontier is lower than the LHG under the group frontier. Finally, according to the above empirical results, this paper puts forward policy suggestions to improve LHG and environmental protection suggestions for laying hens.

## Introduction

Eggs play an irreplaceable role in human survival, production and life^1,2^. As a daily food, they can also be used as raw materials to make cakes, bread and other leisure food. According to the data from Food and Agriculture Organization of the United Nations (FAO), since 1991, China’s egg production has been steadily increasing, with an average annual growth of about 5.07%, and the egg production reached 604.6 billion in 2020. However, it still cannot meet the huge demand of Chinese people for eggs. Therefore, in order to improve the efficiency of layer breeding, this paper will measure the laying hens breeding green total factor productivity (LHG).

In China, during the process of raising laying hens, the basic situation of different scale laying hens will have significant differences^3,4^. Small-scale specialized households, medium-sized farms and large-scale breeding communities are different in many aspects, such as the number of chicken farms, breeding costs, breeding methods, production and breeding income and so on^5–7^. Besides, infrastructure, rural human capital level, breeding technology and environmental governance level will also have different effects due to the diverse scale of laying hens breeding. During the breeding process of different scale, the proportion of each cleaning craft is different, so the chemical oxygen demand (COD), total nitrogen (TN) and total phosphorus (TP) emissions are different^8,9^. It should be pointed out that with the rapid development of laying hens breeding industry, environmental problems are inevitable^10^. According to the second national survey of pollution sources, the annual output of livestock and poultry manure pollution in China reached 3.05 billion tons in 2020. Due to the lack of corresponding facilities for harmless treatment of manure, the surrounding water, soil, air and crops are often polluted^11–13^, and it has become the source of livestock infectious diseases, parasitic diseases and zoonotic diseases^14–17^. Livestock and poultry industry have become one of the main targets of rural environmental pollution control in China^18–20^. Thus, by considering the heterogeneity of scale, this paper uses MinDW-MML model to comprehensively evaluate the change of China’s LHG from 2004 to 2018, which is of great significance to the improvement of China’s laying hens supply and production efficiency. The main reason for choosing MinDW model is that it can overcome the shortcomings of traditional methods that the frontier is too far and frustrates the “enthusiasm” of non-effective production units to catch up.

In this text, the second part describes the situation of related literature. The third part introduces the methodology basis and the data description, mainly including index selection and data sources. Results and discussions are analyzed in the fourth part. Conclusions and policy suggestions are discussed in the fifth part.

## Literature review

At present, the research on laying hens breeding efficiency mainly focuses on the following two aspects.

Firstly, in the research of agricultural production efficiency, there are few studies on laying hens breeding efficiency. Farrell explained the production efficiency from the perspective of input. He believed that the production efficiency is the ratio of the lowest possible cost to the actual production cost when the producer produces some productions with a certain proportion of factor inputs, under the condition that the production technology and market price remain unchanged^21^. Chavas and Aliber estimated the cost efficiency of 545 sample farms of crops and poultry in Wisconsin in 1987. The results showed that the cost efficiency ranged from 0.76 to 0.96^22^. Rowland et al. measured the relative efficiency of 43 pig farms in Kansas in 1988, and found that the characteristics of farmers and farm management methods had different effects on the efficiency^23^. Ameen et al. analyzed the production efficiency of mutton sheep during the period of Spain's accession to the European Union and the EU’s agricultural policy reform. They found that the improvement of mutton sheep production efficiency was the key factor to determine the economic benefit development of Spanish mutton sheep farmers^24^. Shomo et al. calculated the production efficiency of four breeding methods of mutton sheep in arid areas of Syria in 2002, including migration, nomadism, semi-settlement and settlement. The results revealed that the production efficiency of mutton sheep in settlement farming mode was the highest, and that in the migratory mode was the lowest^25^. Tzouramani et al. pointed out that sheep breeding industry in Greece is very essential, especially in the mountainous and semi-mountainous areas. By comparing the production efficiency of organic sheep and conventional sheep, it was found that the production net income of organic sheep was 15.56% higher than that of conventional sheep^26^. Ritter et al. studied the relationship between beef cattle production efficiency and animal welfare. They found most commercial farms gave priority to the high reproductive performance of beef cattle^27^. Zhong et al. studied the production efficiency of pig^28^. Although there are many researches on the production efficiency of livestock and poultry, there are few studies on the efficiency of Chinese laying hens.

The second is the calculation method of laying hens feeding efficiency. With the introduction of production frontier model and the development of production efficiency theory, it has become a trend to use frontier method to study the change of production efficiency. At present, there are mainly two methods for measuring production efficiency. One is the nonparametric method represented by data envelopment analysis (DEA), and the other is the parametric method represented by stochastic frontier analysis (SFA). For the measurement of agricultural efficiency, existing research methods are mostly concentrated on SFA, traditional DEA, Directional Distance Function (DDF) model, and Slack Based Measure (SBM) model. Meeusen and Broeck put forward the SFA method, which is to set the real production inefficiency, using the least square method and the maximum likelihood estimation method to estimate the Cobb–Douglas production function and calculate the production efficiency^29^. Sharma et al. calculated the technical efficiency of Hawaiian pig industry by using DEA model with constant returns to scale and variable returns to scale respectively, but the unexpected output was not considered in the calculation process^30^. Rae et al. used SFA model to estimate the total factor productivity (TFP) of four major livestock and poultry products in China, and found that the development of TFP was relatively slow^31^. Berdikul et al. used SFA model to measure the production efficiency of 69 mutton sheep breeding enterprises in the southeast of the United States. The results showed that the average production technical efficiency of 69 mutton sheep breeding farms was 81%, of which the production efficiency was effective when the breeding scale was 40–60 farms. And the increase of breeding scale would reduce the breeding cost of mutton sheep breeding farms^32^. Although SFA has been widely used, it will produce random measurement error, and its calculation results are easily affected by the selection of influencing factors. Charnes et al. proposed DEA method for the first time, and then many scholars have improved and perfected this method^33^. DEA method is used to evaluate whether the production of each production unit has sufficient production efficiency and study how to improve the inefficient production unit. It can avoid the error caused by the false setting of SFA model, and is more widely used in the study of production efficiency considering environmental constraints. Theodoridis et al. used the traditional DEA method to calculate the production technical efficiency of mutton sheep farmers in Chios island. The results demonstrated that the average production technical efficiency of 58 mutton sheep farmers in Chios island was 0.76, indicating that there was still 24% room for improvement. The larger the scale of breeding, the greater the production efficiency of mutton sheep^34^. Traditional DEA has non-dynamic characteristics. Njuki et al. used the DDF model to calculate the pig breeding efficiency of farms in the eastern United States, and found that the production efficiency of large farms was higher than the production efficiency of small farms^35^. Zhong et al. also used DDF model to evaluate the green total factor productivity of laying hens in China^36^. However, DDF model belongs to radial and angled model, which cannot deal with the problem of relaxation well. Tone proposed SBM model, which design of taking the farthest point from the evaluated unit as the projection point is insufficient^37^. There is a big difference in the index selection of input and undesirable output. And the input indicators fail to reflect the impact of governance investment on environmental performance. Cooper et al. put forward SBM-Undesirable model on this basis^38^. Its advantage is that it not only overcomes the defects of traditional DEA model in strict assumption of radial and angular, but also takes into account the situation of unexpected output and slack variables. However, most decision-making units (DMUs) cannot reach the effective frontier in a short time because the frontier is too far, which is not conducive to the improvement of the overall production efficiency. MinDW model refers to the closest distance between the DMU and the frontier edge, whether the projection point on the frontier edge is strong efficient or weak efficient. Compared with other models, MinDW model has some advantages, making the results more realistic.

Therefore, in order to make up for the shortcomings of the existing research, the innovation of this paper is mainly reflected in the following three aspects. (1) There are few literatures about laying hens feeding. This paper studies the feeding efficiency of laying hens in China, which is innovative. (2) In terms of research methods, this paper constructs MinDW model and MML index considering scale heterogeneity to evaluate LHG with different scales. (3) In this paper, the environmental factors are introduced into the evaluation system of laying hens breeding efficiency, and the negative output is taken into account when selecting the index, that is, the environmental pollution of laying hens breeding industry. Based on the above innovation points, this paper finally puts forward policy suggestions to improve China’s LHG and environmental protection suggestions for laying hens breeding.

## Methodology

### Minimum distance to weak efficient frontier

Briec and Charnes et al. first proposed the Minimum distance to weak efficient frontier (MinDW) model^39,40^, which can be expressed as $m + n$ linear programming ($m$ is the number of input indicators and $n$ is the number of output indicators), assuming that the input variable is $x$ and the output variable is $y$. The specific formula is shown in Eq. (1):

1 $$ \begin{aligned} & \max \beta_{z} ,z = 1,2, \ldots ,m + n \\ & s.t.\left\{ \begin{gathered} \sum\nolimits_{j = 1}^{q} {\alpha_{j} x_{rj} + \beta_{z} e_{r} \le x_{rk} ,r = 1,2, \ldots ,m} \hfill \\ \sum\nolimits_{j = 1}^{q} {\alpha_{j} x_{ij} + \beta_{z} e_{i} \ge y_{ik} ,i = 1,2, \ldots ,n} \hfill \\ \alpha_{j} \ge 0 \hfill \\ \end{gathered} \right. \\ \end{aligned} $$

$e_{r}$ and $e_{i}$ are constants. In the programming formula, only one $e$ is equal to 1, and the others are 0, that is shown in Eq. (2):

2 $$ \begin{aligned} & e_{r} = 1\;{\text{ if}}\; \, r = z; \, e_{r} = 0 \, \;{\text{if}}\; \, r \ne z \\ & e_{i} = 1 \, \;{\text{if}}\; \, i = z - m; \, e_{r} = 0 \, \;{\text{if}}\; \, i \ne z - m \\ \end{aligned} $$

The efficiency value of model is expressed as Eq. (3):

3 $$ \theta_{z}^{*} = \frac{{1 - \frac{1}{m}\sum\nolimits_{r = 1}^{m} {\frac{{\beta_{z}^{*} e_{r} }}{{x_{rk} }}} }}{{1 + \frac{1}{n}\sum\nolimits_{i = 1}^{n} {\frac{{\beta_{z}^{*} e_{i} }}{{y_{ik} }}} }} $$

The efficiency value of MinDW model is expressed as $\theta_{\max }^{*} = \max (\theta_{z}^{*} ,z = 1,2, \cdots ,m + n)$, and the maximum efficiency value corresponds to the minimum $\beta^{*}$, that is the nearest distance to the frontier.

This paper uses the MinDW model with negative output to conduct empirical analysis. The method can be expressed as $m + n + d$ linear programming ($m$ is the number of inputs, $n$ is the number of desirable output, $d$ is the number of unexpected output), assuming that the input variable is $x$, the desirable output variable is $y$, and the undesirable output variable is $f$. The specific formula is shown in Eq. (4):

4 $$ \begin{aligned} & \max \beta_{z} ,z = 1,2, \ldots ,m + n + d \\ & s.t.\left\{ \begin{gathered} \sum\nolimits_{j = 1}^{q} {\alpha_{j} x_{rj} + \beta_{z} e_{r} \le x_{rk} ,r = 1,2, \ldots ,m} \hfill \\ \sum\nolimits_{j = 1}^{q} {\alpha_{j} x_{ij} - \beta_{z} e_{i} \ge y_{ik} ,i = 1,2, \ldots ,n} \hfill \\ \sum\nolimits_{j = 1}^{q} {\alpha_{j} x_{lj} + \beta_{z} e_{l} \le f_{lk} ,l = 1,2, \ldots ,d} \hfill \\ \alpha_{j} \ge 0 \hfill \\ \end{gathered} \right. \\ \end{aligned} $$

$e_{r}$, $e_{i}$ and $e_{l}$ are constants. In the programming formula, only one $e$ is equal to 1, and the others are 0, that is shown in Eq. (5):

5 $$ \begin{aligned} & e_{r} = 1\;{\text{ if}}\; \, r = z; \, e_{r} = 0 \, \;{\text{if}}\; \, r \ne z \\ & e_{i} = 1 \, \;{\text{if }}\;i = z - m; \, e_{r} = 0 \, \;{\text{if}}\; \, i \ne z - m \\ & e_{l} = 1 \, \;{\text{if}}\; \, l = z - m - n; \, e_{l} = 0 \, \;{\text{if}}\; \, l \ne z - m - n \\ \end{aligned} $$

The efficiency value of model is expressed as Eq. (6):

6 $$ \theta_{z}^{*} = \frac{{1 - \frac{1}{m}\sum\nolimits_{r = 1}^{m} {\frac{{\beta_{z}^{*} e_{r} }}{{x_{rk} }}} }}{{1 + \frac{1}{n + d}\left( {\sum\nolimits_{i = 1}^{n} {\frac{{\beta_{z}^{*} e_{i} }}{{y_{ik} }}} + \sum\nolimits_{l = 1}^{d} {\frac{{\beta_{z}^{*} e_{l} }}{{f_{lk} }}} } \right)}} $$

The efficiency value of MinDW model is expressed as $\theta_{\max }^{*} = \max (\theta_{z}^{*} ,z = 1,2, \cdots ,m + n + d)$, and the maximum efficiency value corresponds to the minimum $\beta^{*}$, which means the nearest distance to the frontier.

The efficiency value of MinDW model will not be less than the efficiency value of directional distance function model with any direction vector or other distance types (such as radial model and SBM model). In other words, the efficiency value of MinDW model is the largest. Combined with the above process, we can define the common boundary ($\beta^{meta*}$) and the model is as Eq. (7):

7 $$ \begin{aligned} & \beta^{meta*} = \max \frac{{1 - \frac{1}{m}\sum\nolimits_{r = 1}^{m} {\frac{{\beta_{z} e_{r} }}{{x_{rk} }}} }}{{1 + \frac{1}{n + d}\left( {\sum\nolimits_{i = 1}^{n} {\frac{{\beta_{z} e_{i} }}{{y_{ik} }}} + \sum\nolimits_{l = 1}^{d} {\frac{{\beta_{z} e_{l} }}{{f_{lk} }}} } \right)}} \\ & s.t.\left\{ \begin{gathered} \sum\nolimits_{j = 1}^{{q_{m} }} {\alpha_{j} x_{rj} + \beta_{z} e_{r} \le x_{rk} ,r = 1,2, \cdots ,m} \hfill \\ \sum\nolimits_{j = 1}^{{q_{m} }} {\alpha_{j} x_{ij} - \beta_{z} e_{i} \ge y_{ik} ,i = 1,2, \cdots ,n} \hfill \\ \sum\nolimits_{j = 1}^{{q_{m} }} {\alpha_{j} x_{lj} + \beta_{z} e_{l} \le f_{lk} ,l = 1,2, \cdots ,d} \hfill \\ \alpha_{j} \ge 0 \hfill \\ \end{gathered} \right. \\ \end{aligned} $$

Similarly, the efficiency value of DMU relative to the scale frontier ($\beta^{scale*}$) can be obtained by the Eq. (8):

8 $$ \begin{aligned} & \beta^{scale*} = \max \frac{{1 - \frac{1}{m}\sum\nolimits_{r = 1}^{m} {\frac{{\beta_{z} e_{r} }}{{x_{rk} }}} }}{{1 + \frac{1}{n + d}\left( {\sum\nolimits_{i = 1}^{n} {\frac{{\beta_{z} e_{i} }}{{y_{ik} }}} + \sum\nolimits_{l = 1}^{d} {\frac{{\beta_{z} e_{l} }}{{f_{lk} }}} } \right)}} \\ & s.t.\left\{ \begin{gathered} \sum\nolimits_{j = 1}^{{q_{s} }} {\alpha_{j} x_{rj} + \beta_{z} e_{r} \le x_{rk} ,r = 1,2, \ldots ,m} \hfill \\ \sum\nolimits_{j = 1}^{{q_{s} }} {\alpha_{j} x_{ij} - \beta_{z} e_{i} \ge y_{ik} ,i = 1,2, \ldots ,n} \hfill \\ \sum\nolimits_{j = 1}^{{q_{s} }} {\alpha_{j} x_{lj} + \beta_{z} e_{l} \le f_{lk} ,l = 1,2, \ldots ,d} \hfill \\ \alpha_{j} \ge 0 \hfill \\ \end{gathered} \right. \\ \end{aligned} $$

Finally, in the common frontier model, the technology gap ratio (TGR) is equal to the ratio of the efficiency value of the common frontier to the scale frontier^41^. The formula is as Eq. (9):

9 $$ TGR^{MinDW} = \frac{{\beta^{meta*} }}{{\beta^{scale*} }} $$

$\beta^{meta*}$ and $\beta^{scale*}$ represent the optimal solution of formula (7) and formula (8), respectively. Obviously, $0 \le TGR \le 1$. TGR is used to measure the distance between the optimal production technology and the potential optimal technology of a group, and identify whether there are any differences in LHG under different groups. The closer the TGR is to 1, the closer the technology level is to the optimal potential technology level. Conversely, it shows the larger gap between the technology level and the potential optimal technology level.

### Metafrontier-Malmquist–Luenberger index

Malmquist productivity index is widely used in the study of dynamic efficiency change trend, and has good adaptability to multiple input–output data and panel data analysis. The actual production process often contains unexpected output. After Chung et al. proposed Malmquist–Luenberger (ML) index, any Malmquist index with undesired output can be called ML index^42^. Oh constructed the Global-Malmquist–Luenberger index^43^. All the evaluated DMUs are included in the global reference set, which avoids the phenomenon of infeasible solution in VRS. The global reference set constructed in this paper is as Eqs. (10)–(11):

10 $$ Q^{G} \left( x \right) = Q^{1} \left( {x^{1} } \right) \cup Q^{2} \left( {x^{2} } \right) \cup \cdots \cup Q^{T} \left( {x^{T} } \right) $$

11 $$ Q^{t} \left( {x^{t} } \right) = \left\{ {\left( {y^{t} ,f^{t} } \right)\left| {x^{t} \;can\;produce} \right.\;\left( {y^{t} ,f^{t} } \right)} \right\} $$

This paper takes MML index as the LHG.

12 $$ \begin{aligned} MML_{t - 1}^{t} & = \sqrt {\frac{{1 - D_{t - 1} \left( {x^{t} ,y^{t} ,f^{t} ;y^{t} , - f^{t} } \right)}}{{1 - D_{t - 1} \left( {x^{t - 1} ,y^{t - 1} ,f^{t - 1} ;y^{t - 1} , - f^{t - 1} } \right)}} \times \frac{{1 - D_{t} \left( {x^{t} ,y^{t} ,f^{t} ;y^{t} , - f^{t} } \right)}}{{1 - D_{t} \left( {x^{t - 1} ,y^{t - 1} ,f^{t - 1} ;y^{t - 1} , - f^{t - 1} } \right)}}} \\ & = \sqrt {\frac{{1 - D_{t - 1} \left( {x^{t - 1} ,y^{t - 1} ,f^{t - 1} ;y^{t - 1} , - f^{t - 1} } \right)}}{{1 - D_{t} \left( {x^{t - 1} ,y^{t - 1} ,f^{t - 1} ;y^{t - 1} , - f^{t - 1} } \right)}} \times \frac{{1 - D_{t - 1} \left( {x^{t} ,y^{t} ,f^{t} ;y^{t} , - f^{t} } \right)}}{{1 - D_{t} \left( {x^{t} ,y^{t} ,f^{t} ;y^{t} , - f^{t} } \right)}}} \\ & \;\;\;\;\; \times \frac{{1 - D_{t} \left( {x^{t} ,y^{t} ,f^{t} ;y^{t} , - f^{t} } \right)}}{{1 - D_{t - 1} \left( {x^{t - 1} ,y^{t - 1} ,f^{t - 1} ;y^{t - 1} , - f^{t - 1} } \right)}} \\ \end{aligned} $$

Next, it further decompose the MML index into efficiency change (EC) and technology change (TC). The specific formula is shown in Eqs. (13)–(14):

13 $$ TC_{t - 1}^{t} = \sqrt {\frac{{1 - D_{t - 1} \left( {x^{t - 1} ,y^{t - 1} ,f^{t - 1} ;y^{t - 1} , - f^{t - 1} } \right)}}{{1 - D_{t} \left( {x^{t - 1} ,y^{t - 1} ,f^{t - 1} ;y^{t - 1} , - f^{t - 1} } \right)}} \times \frac{{1 - D_{t - 1} \left( {x^{t} ,y^{t} ,f^{t} ;y^{t} , - f^{t} } \right)}}{{1 - D_{t} \left( {x^{t} ,y^{t} ,f^{t} ;y^{t} , - f^{t} } \right)}}} $$

14 $$ EC_{t - 1}^{t} = \frac{{1 - D_{t} \left( {x^{t} ,y^{t} ,f^{t} ;y^{t} , - f^{t} } \right)}}{{1 - D_{t - 1} \left( {x^{t - 1} ,y^{t - 1} ,f^{t - 1} ;y^{t - 1} , - f^{t - 1} } \right)}} $$

where $\left( {x^{t - 1} ,y^{t - 1} ,f^{t - 1} } \right)$ and $\left( {x^{t} ,y^{t} ,f^{t} } \right)$ represent the input, expected output and unexpected output of t-1 and t, respectively. $TC_{t - 1}^{t}$ is the devotion to LHG raise of DMU’s technical progress from $t - 1$ to $t$. And $EC_{t - 1}^{t}$ represents the devotion to LHG raise of DMU's efficiency improvement from $t - 1$ to $t$. The higher the value is, the larger the devotion is. The $MML$ index is recorded as $MI$. The value of $MI$ is the LHG. The green total factor productivity index of laying hens breeding under the common frontier and scale frontier are as Eqs. (15)–(16):

15 $$ metaMI_{t - 1}^{t} = \sqrt {\frac{{1 - D_{{_{t - 1} }}^{m} \left( {x^{t} ,y^{t} ,f^{t} ;y^{t} , - f^{t} } \right)}}{{1 - D_{{_{t - 1} }}^{m} \left( {x^{t - 1} ,y^{t - 1} ,f^{t - 1} ;y^{t - 1} , - f^{t - 1} } \right)}} \times \frac{{1 - D_{{_{t} }}^{m} \left( {x^{t} ,y^{t} ,f^{t} ;y^{t} , - f^{t} } \right)}}{{1 - D_{{_{t} }}^{m} \left( {x^{t - 1} ,y^{t - 1} ,f^{t - 1} ;y^{t - 1} , - f^{t - 1} } \right)}}} $$

16 $$ groupMI_{t - 1}^{t} = \sqrt {\frac{{1 - D_{{_{t - 1} }}^{g} \left( {x^{t} ,y^{t} ,f^{t} ;y^{t} , - f^{t} } \right)}}{{1 - D_{{_{t - 1} }}^{g} \left( {x^{t - 1} ,y^{t - 1} ,f^{t - 1} ;y^{t - 1} , - f^{t - 1} } \right)}} \times \frac{{1 - D_{{_{t} }}^{g} \left( {x^{t} ,y^{t} ,f^{t} ;y^{t} , - f^{t} } \right)}}{{1 - D_{{_{t} }}^{g} \left( {x^{t - 1} ,y^{t - 1} ,f^{t - 1} ;y^{t - 1} , - f^{t - 1} } \right)}}} $$

For the DMUs with scale heterogeneity, we can measure the technology gap between the group frontier and the common frontier, which is caused by the specific group structure.

### Data and variables

Based on the research of the existing literature^36^, this paper selects five indexes to build the input–output indicator system. Details are as below:

1. Input variables:
  1. Quantity of concentrated forage. Mainly includes seeds of crops and their by-products.
  2. Quantity of grain consumption. Quantity of grain consumed is the quantity of grain consumed by laying hens when they are raised. For example: corn, sorghum, broken rice, wheat, barley, wheat bran, etc.
  3. Material expenses. The sum of water and fuel power costs, labor costs, and medical epidemic prevention fees. Water and fuel power costs include water, electricity, coal and other fuel power costs; labor costs mean the human management cost of each laying hen from the brood stage to the laying stage; medical and epidemic prevention costs include the cost of disease prevention and control.
2. *Positive output* Main product production, which is the egg production per layer.
3. *Negative output* Total discharge. According to the calculation method of *The Manual of Pollutant Discharge Coefficient*, Eq. (17) is used to calculate the COD, TN, and the TP of each layer. Then, according to the calculation method of class GB3838-2002 water quality standard in V, Eq. (18) is used to calculate the total discharge.

17 $$ POLLUTANTS = FP(FD) \times Days $$

18 $$ TOTAL \, POLLUTANTS = \frac{COD}{{40}} + \frac{TN}{2} + \frac{TP}{{0.4}} $$

where, $FP(FD)$ is the pollution discharge coefficient and the $Days$ is the average raising days. Descriptive statistics of input and output indicators are shown in Table 1.

**Table 1:** Descriptive statistics of input and output indicators.

Criterion layer	Index	Unit	Scale	Max	Min	Mean	Std. dev	Obs
Input	Concentrated forage	kg	Small	55.63	34.05	41.23	3.63	105
			Middle	48.92	20.40	39.89	4.63	315
			Large	51.44	21.23	40.02	4.59	270
	Grain consumption	kg	Small	41.72	23.83	29.30	2.89	105
			Middle	43.64	17.23	28.45	3.60	315
			Large	128.17	13.80	29.06	7.19	270
	Material expenses	yuan	Small	25.36	3.64	11.88	5.98	105
			Middle	21.73	2.41	9.56	4.36	315
			Large	39.30	15.24	9.75	5.27	270
Positive output	Main product production	kg	Small	18.87	13.27	16.94	1.25	105
			Middle	19.97	10.68	16.99	1.46	315
			Large	20.53	8.50	17.12	17.39	270
Negative output	Total discharge	kg	Small	0.59	0.26	0.47	0.09	105
			Middle	0.59	0.18	0.41	0.15	315
			Large	0.76	0.12	0.44	0.20	270

The quantity of concentrate, the quantity of food consumed, the cost of labor, the cost of medical treatment all come from “National Agricultural Product Cost and Benefit Data Compilation”. The pollutant discharge coefficient of laying hens is derived from “The Manual of Pollutant Discharge Coefficient”. According to the definition of scale in above two materials, a small scale 300–1000 laying hens, a medium scale 1000–10,000 laying hens, and a large scale greater than 10,000 laying hens are grouped to calculate cost efficiency.

From 2004 to 2018, this paper selects 24 major egg-producing provinces (municipalities) in China as samples, after eliminating singular data in the three scales and averaging the missing data, the final small-scale group is left with 7 provinces including Liaoning, Shandong, Henan, Heilongjiang, Jilin, Shanxi, and Shaanxi; the medium-scale group is the remaining 21 provinces of Beijing, Hebei, Jiangsu, Liaoning, Shandong, Tianjin, Zhejiang, Anhui, Henan, Heilongjiang, Jilin, Hubei, Inner Mongolia, Shanxi, Yunnan, Gansu, Ningxia, Shaanxi, Sichuan, Xinjiang, Chongqing; the large-scale group has 18 provinces, including Beijing, Fujian, Guangdong, Henan, Jiangsu, Liaoning, Shandong, Tianjin, Anhui, Henan, Heilongjiang, Hubei, Jilin, Shanxi, Yunnan, Gansu, Sichuan and Chongqing.

As is shown in Table 2, after dividing the provinces by region, the eastern region has 10 provinces (municipalities): Liaoning, Shandong, Beijing, Hebei, Jiangsu, Tianjin, Zhejiang, Fujian, Guangdong, Henan. The central region has 7 provinces (autonomous region): Henan, Heilongjiang, Jilin, Shanxi, Anhui, Hubei, Inner Mongolia. The western region has 7 provinces (municipalities): Shaanxi, Gansu, Ningxia, Sichuan, Xinjiang, Chongqing, Yunnan.

**Table 2:** Samples selected from 2004–2018.

	Small scale	Medium scale	Large scale
Eastern region	Liaoning, Shandong	Beijing, Hebei, Jiangsu, Liaoning, Shandong, Tianjin, Zhejiang	Beijing, Fujian, Guangdong, Henan, Jiangsu, Liaoning, Shandong, Tianjin
Central region	Henan, Heilongjiang, Jilin, Shanxi	Anhui, Henan, Heilongjiang, Jilin, Hubei, Inner Mongolia, Shanxi	Anhui, Henan, Heilongjiang, Hubei, Jilin, Shanxi
Western region	Shaanxi	Gansu, Ningxia, Shaanxi, Sichuan, Xinjiang, Chongqing, Yunnan	Gansu, Sichuan, Chongqing, Yunnan

## Results and discussions

This paper measures China’s LHG in three different scales from 2004 to 2018, and then it analyzes the evolution trend and characteristics of LHG.

### China’s temporal dynamic change of LHG

**Figure 1:**
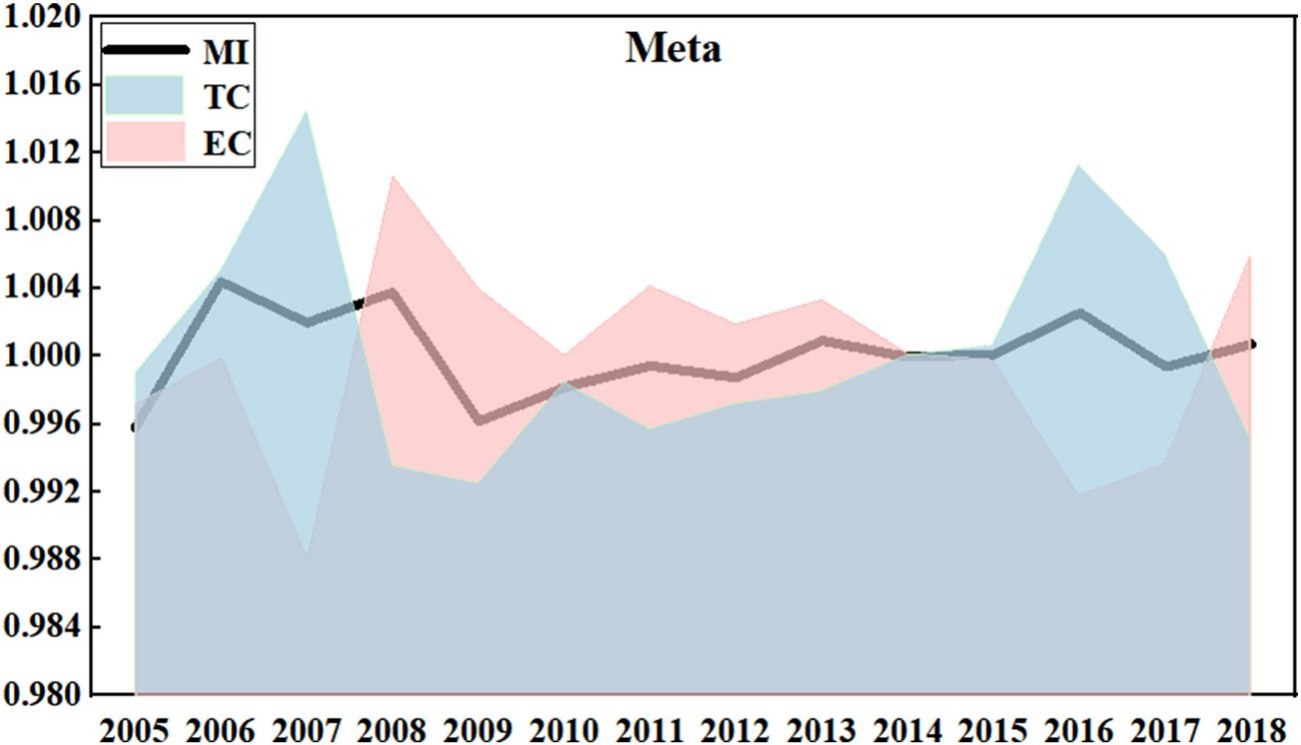
LHG and its decomposition index under meta-frontier from 2004–2018.

Figure 1 shows the comprehensive LHG of three scales and its decomposition indicators (TC and EC) under the common frontier. Under the common frontier, China’s LHG has been fluctuating around 1, with a large fluctuation before 2009 and a relatively small fluctuation after 2009. The maximum value of these 15 years appeared in 2006 (1.0044), and the minimum value occurred in 2005 (0.9958). This is mainly because before 2005, China's laying hens feeding industry lacks sufficient environmental awareness. Since 2006, the profit of laying hens breeding has increased, making the enthusiasm of farmers gradually improve. Since 2008, the laying hens’ industry has entered a stage of self-integration, and the impact of the “avian influenza” incident has accelerated this process. At present, specialized family farming and small-sized breeding are the main forms of laying hens breeding. The larger the scale of breeding, the higher the input rate of professional equipment and technology. Since the early 1970s, China has introduced automatic breeding technology and equipment from abroad. After nearly 30 years of development, China's automatic breeding technology has formed a scale, but there is still a certain gap with foreign countries. In the future, China should strengthen the input in clean farming technology. From 2009 to 2016, it showed an upward trend on the whole. The outbreak of H7N9 epidemic in the first half of 2017, the depressed market and the rapid turnover of the market in the second half of 2017 caused heavy losses to farmers and forced a large number of farms to close, which accelerated the transformation of China’s laying hens feeding industry pattern from “small scale, large group” to “medium scale, medium group”. In 2018, China's LHG was 1.0007, which increased by 0.07%. Although the growth value is small, the positive growth also indicates a good trend.

**Figure 2:**
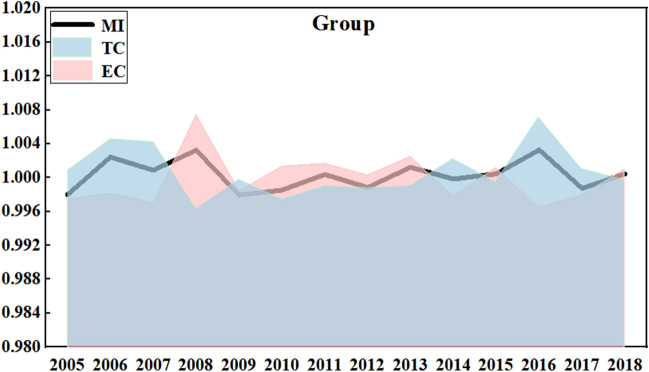
LHG and its decomposition index under group frontier from 2004–2018.

The comprehensive LHG of three scales from 2004 to 2018, under the group frontier (scale frontier), is shown in Fig. 2. The fluctuation trend of LHG in group frontier is the same as that in common frontier, but the peak value is different. Under the group frontier, it reached a small peak (1.0032) in 2008 and a total peak (1.0033) in 2016. In 2009, LHG was the lowest (0.9979) in recent 15 years. Obviously, China’s laying hens feeding industry is facing some constraints, such as resources, technology, economy and environment. For example, due to the lack of effective digestion and treatment of feces and sewage, environmental problems are becoming more and more prominent. It should note that environmental problems have become the main bottleneck of the development of China’s livestock and poultry industry. Thus, how to solve the difficult problem between large-scale breeding and environmental protection has become one of the keys to the sustainable development of China’s laying hens feeding industry. The government and relevant departments should actively seek the healthy and sustainable development path of laying hens breeding industry, and strengthen the construction of supporting facilities and personnel training. According to the average data over the past 15 years, the MI, EC and TC under the common frontier are 1.00013, 0.9999 and 1.00025, respectively, while the MI, EC and TC under the group frontier are 1.00029, 0.99989 and 1.00045, respectively. The results obtained under the common frontier and the group frontier are different, because the group frontier constructs the frontier surface based on the different scale of the laying hens breeding, while the common frontier builds the frontier surface based on all sizes of the laying hens breeding. Obviously, the LHG is significantly overestimated under the group frontier.

**Figure 3:**
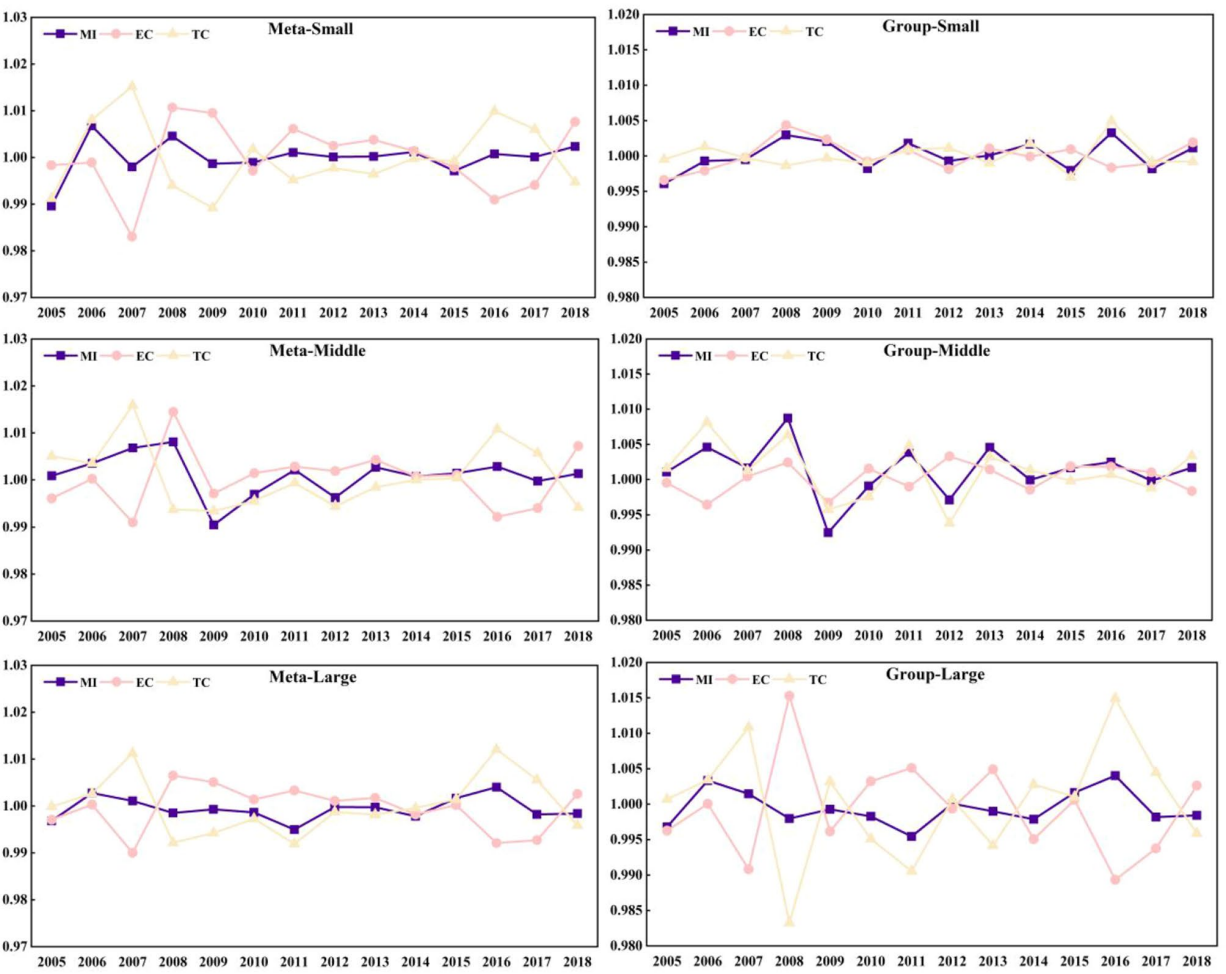
Three-sized LHG and its decomposition index from 2004 to 2018.

As shown in Fig. 3, the trend of LHG in different scales is basically the same under the common frontier and the group frontier. Under the common frontier, the average LHG of small-large, medium-large and large-scale are 0.99997, 1.00100 and 0.99942 respectively. Under the group frontier, they are 1.00011, 1.00134 and 0.99942 respectively. Small-scale LHG is lower than 1 under the common frontier and higher than 1 under the group frontier, this result fully illustrates the scientificity and necessity of considering scale heterogeneity in the analysis of LHG. Middle-scaled LHG has the largest fluctuation, but the overall level is the highest, and it has a positive growth. Then, the second is small scale, and the last is large scale. The large-scale performance is relatively stable, because the large-scale laying hens feeding is easily affected by the policies and technologies. Large-scale laying hens have a strong ability to deal with pollutants. However, due to the large amount of total emissions, there are certain strict requirements for equipment, labor force and professional technical level. Therefore, large-scale laying hens show the coexistence of high-tech level and low resource utilization rate. It should note that the larger scale does not mean more advantageous growth for LHG. From the perspective of growth trend, environmental constraints have a greater impact on China's laying hens feeding industry, and the growth rate of LHG has slowed down comprehensively.

The development trend of feed demand for different scaled laying hens is quite different, and there are also differences in factor input, cost–benefit, domestic employment and hired workers. Due to the diversities in production factor input, there are differences in technical efficiency, cost efficiency and efficiency influencing factors among different scale farmers. In addition, for different scale farmers, some differences exist in the input of pollution control, willingness and level of environmental protection payment, and ways of manure treatment. The production capacity and technical level of small-scale farmers cannot meet the needs of laying hens breeding and egg consumption. Especially under the background of internationalization, it has become an inevitable trend for the development of laying hens feeding industry to transform from small-scale breeding to middle-scale and large-scale standardized breeding.

**Figure 4:**
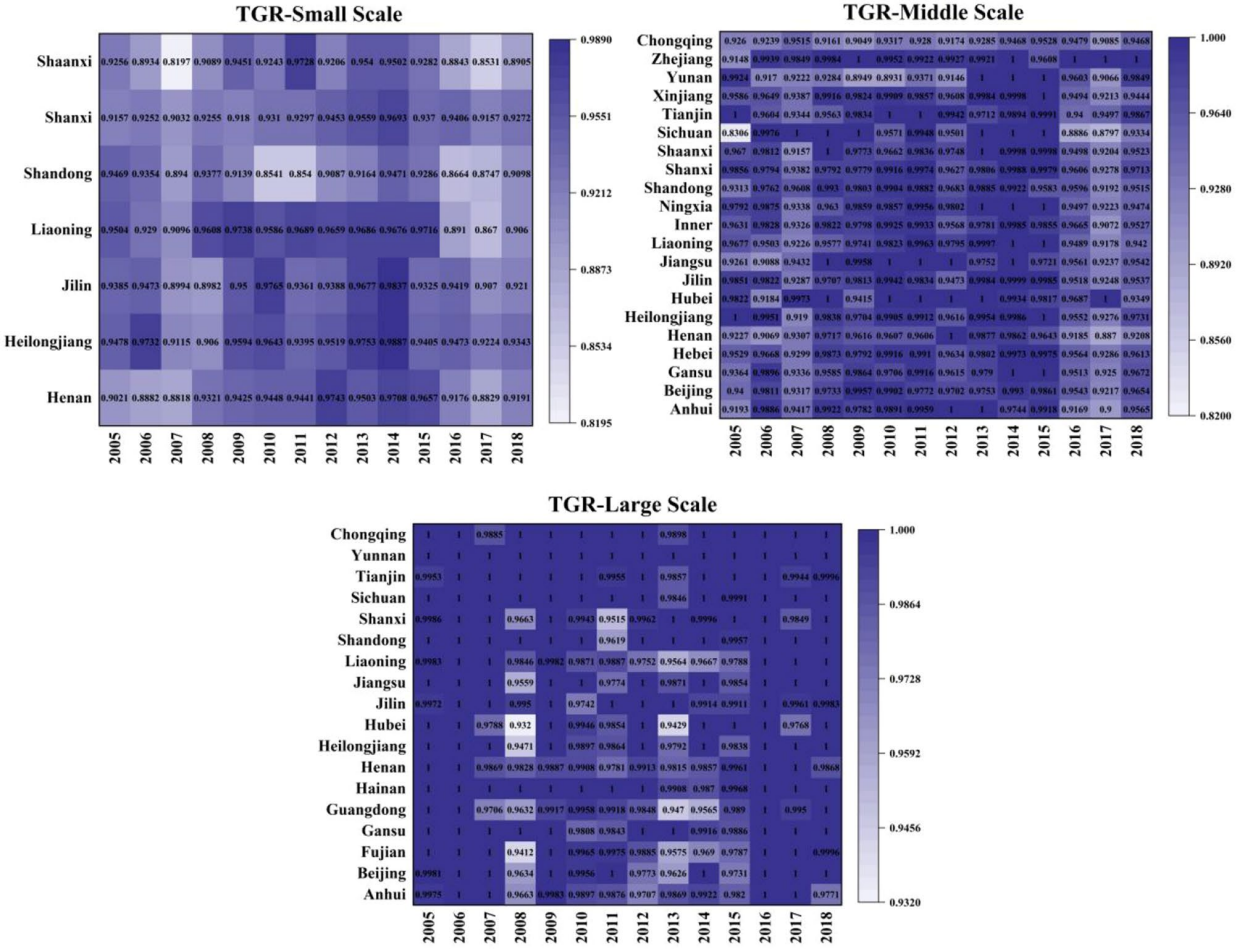
Three-sized TGR from 2004–2018.

As shown in Fig. 4, the technology gap ratio is used to measure the distance between the optimal production scale and the potential optimal scale of the group. The closer the ratio is to 1, the closer the scale level is to the optimal potential scale level. On the contrary, the farther the ratio is to 1, the greater the gap between the scale level and the potential optimal scale level. Large-scale TGR is closest to 1, followed by middle-scale and finally small-scale. It suggests that large-scale is suitable for the development trend of laying hens breeding industry in China. At present, the main reason for the loss of production efficiency of laying hens breeding farmers in China is the low efficiency of factor allocation. By promoting the reform of land market, labor market and capital market in rural areas, it is obvious that laying hens farmers can better allocate resources. In China, nearly 90% of the large-scale laying hens breeding farms have not undergone the environmental impact assessment, 60% of the farms lack the necessary pollution treatment measures, and the level of waste utilization and treatment is low. The pollution from the laying hens feeding industry has become the main source of non-point source pollution in rural areas. Therefore, in order to reduce pollution and improve the efficiency of laying hens breeding, it is necessary to gradually shift from small-scale breeding to middle-scale and large-scale breeding in the future, and give full play to the scale effect.

### China’s spatial change of LHG

**Figure 5:**
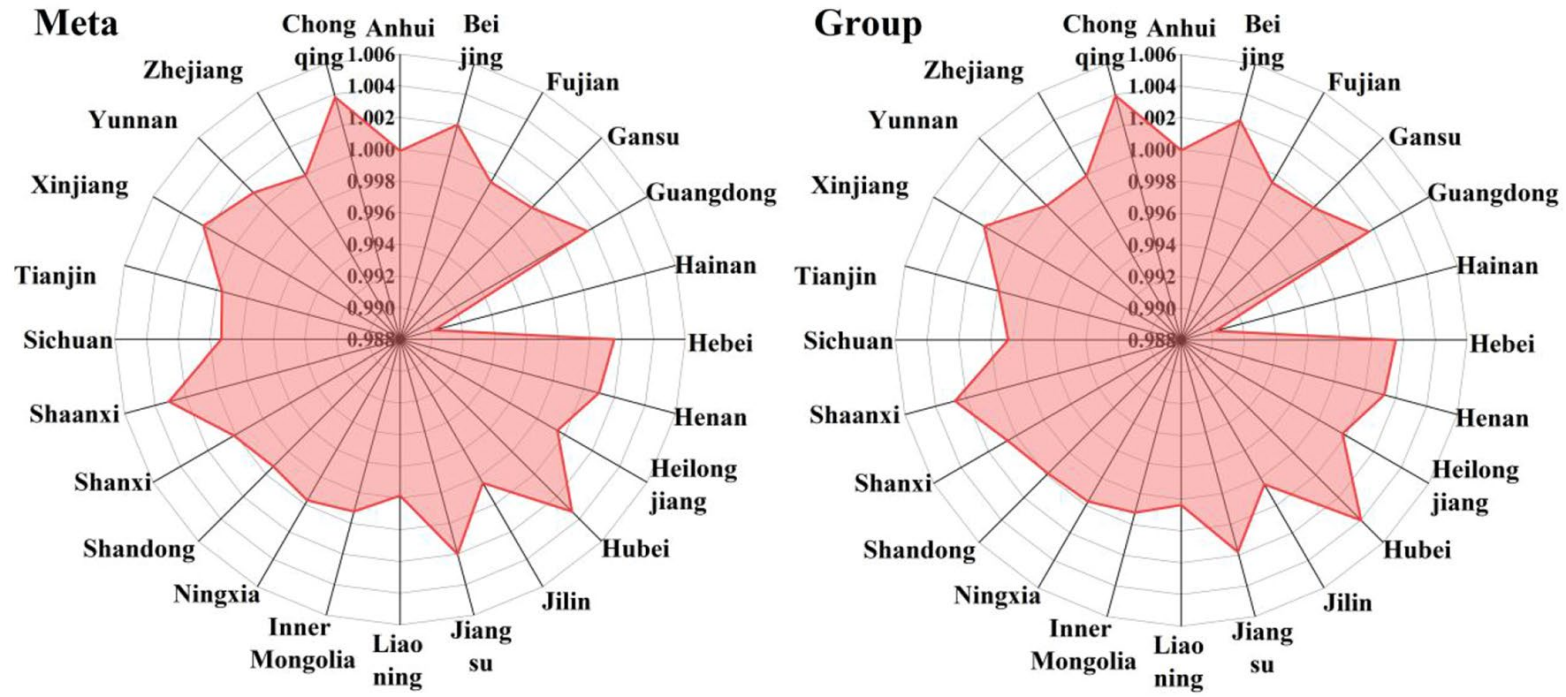
Average LHG in each province.

The combined LHG of three scales under the common frontier and scale frontier is shown in Fig. 5. In general, LHG under the group frontier is higher than the LHG under the common frontier. From the perspective of a single province, the LHGs of Chongqing (1.00382), Hubei (1.00335), Shaanxi (1.00310), Xinjiang (1.00233) and Beijing (1.00202) are relatively high under the common frontier, while LHGs of Hubei (1.00403), Chongqing (1.00393), Shaanxi (1.00274), Beijing (1.00234) and Xinjiang (1.00233) are relatively high under the group frontier. In 24 provinces, the top five provinces under the meta-frontier and the group frontier are the same. With the acceleration of urbanization and industrialization, a large number of rural labor force have shifted from the countryside to the city, from the primary industry to the secondary industry and the tertiary industry. The laying hens feeding behavior of breeders will not only be affected by the comparative interests within the animal husbandry, but also by the non-agricultural employment opportunities. At present, China's rural laying hens breeding technology is backward, capital turnover is not smooth, herd mentality is serious, and lack of market control, which is difficult to form a brand effect. Moreover, a series of problems, such as the difficult examination and approval of breeding land, the high cost of breeding in the early stage of laying, the lack of professional breeding talents, and the difficult environmental pollution prevention and control, have become the bottleneck of laying hens breeding. The main reasons for the low LHG in China are the low overall efficiency and the large technical gap between provinces. Therefore, improving the efficiency level and narrowing the technology gap between regions are effective ways to improve LHG.

**Figure 6:**
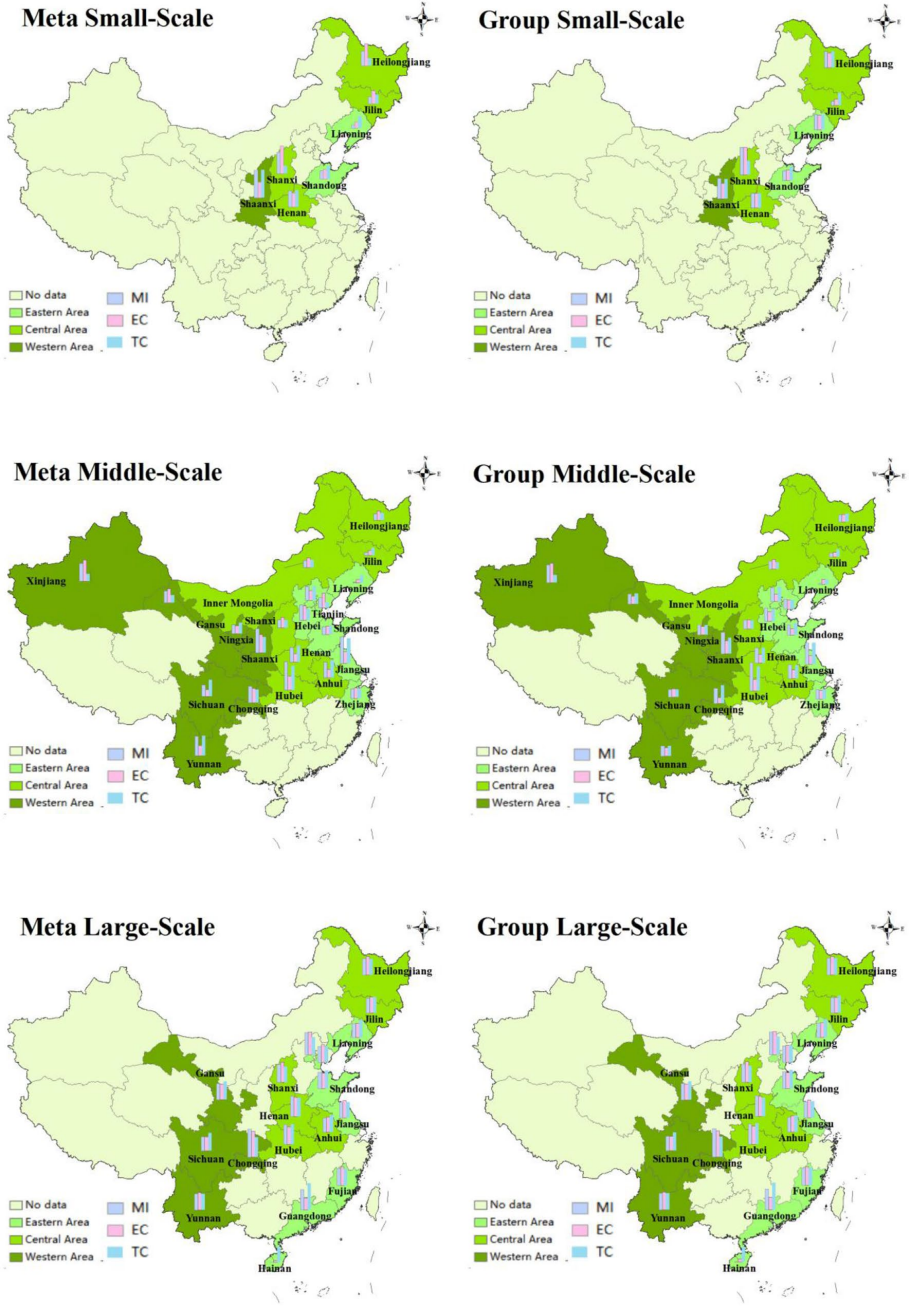
Three-sized average LHG and its decomposition index in different regions. *Source* ArcMap10.7 https://desktop.arcgis.com/zh-cn/arcmap/.

The situation of different scales laying hens in different areas is shown in Fig. 6. Overall, LHG is the highest in the western region, followed by the central region, and the lowest in the eastern region. The LHG of western region, central region and eastern region were 1.00130, 1.00021 and 0.99936 respectively under the common frontier, and the LHG of three regions under the group frontier were 1.00105, 1.00047 and 0.99950 respectively. The LHG of three scales in the western region is the highest. The main reason for the high LHG in the western region is the improvement of farm facilities and the application and popularization of information technology, which greatly improves the LHG. In addition, the central region, with superior geographical position, is located in the middle of China. Obviously, transportation condition is an important factor affecting the distribution of laying hens breeding industry. On the one hand, the improvement of transportation and infrastructure conditions makes it easier for laying hens to adopt new technologies, promote technological progress and economic growth, improve laying hens’ productivity, and stimulate production enthusiasm. On the other hand, the improvement of transportation facilities can also provide more convenient conditions for local laying hens sales, improve the input–output ratio of factors, and promote the increase of production efficiency, thus influencing the decision-making behavior of breeders. Furthermore, the eastern region has a high level of economic development, dense population and limited geographical space, which is not suitable for the development of livestock and poultry breeding. Finally, LHG showed an upward trend under the common frontier and group frontier, which indicates the overall development of China’s laying hens breeding industry is optimistic.

Under the common frontier, the average LHG of small-scale, medium-scale and large-scale in China is 0.99997, 1.00100 and 0.99942 respectively, the EC is 1.00017, 1.00033 and 0.99946 respectively, and the TC is 0.99990, 1.00077 and 1.00007 respectively. Under the group frontier, the average LHG of three scales is 1.00011, 1.00134 and 0.99942 respectively, the EC is 1.00004, 1.00017 and 0.99948 respectively, and the TC is 1.00008, 1.00118 and 1.00008 respectively. The result reflects efficiency decline and technological progress. Under the background of the deepening of socialist market economy, the breeding mode of “small scale, large group” is no longer in line with China’s national conditions, and moderately large-scale breeding will become the mainstream of the industry. Compared with the traditional small-scale breeding, moderate scale breeding can effectively reduce the cost and resist market risk, which meets the needs of modern agricultural development. Therefore, medium-sized LHG is higher than small-scale and large-scale.

**Figure 7:**
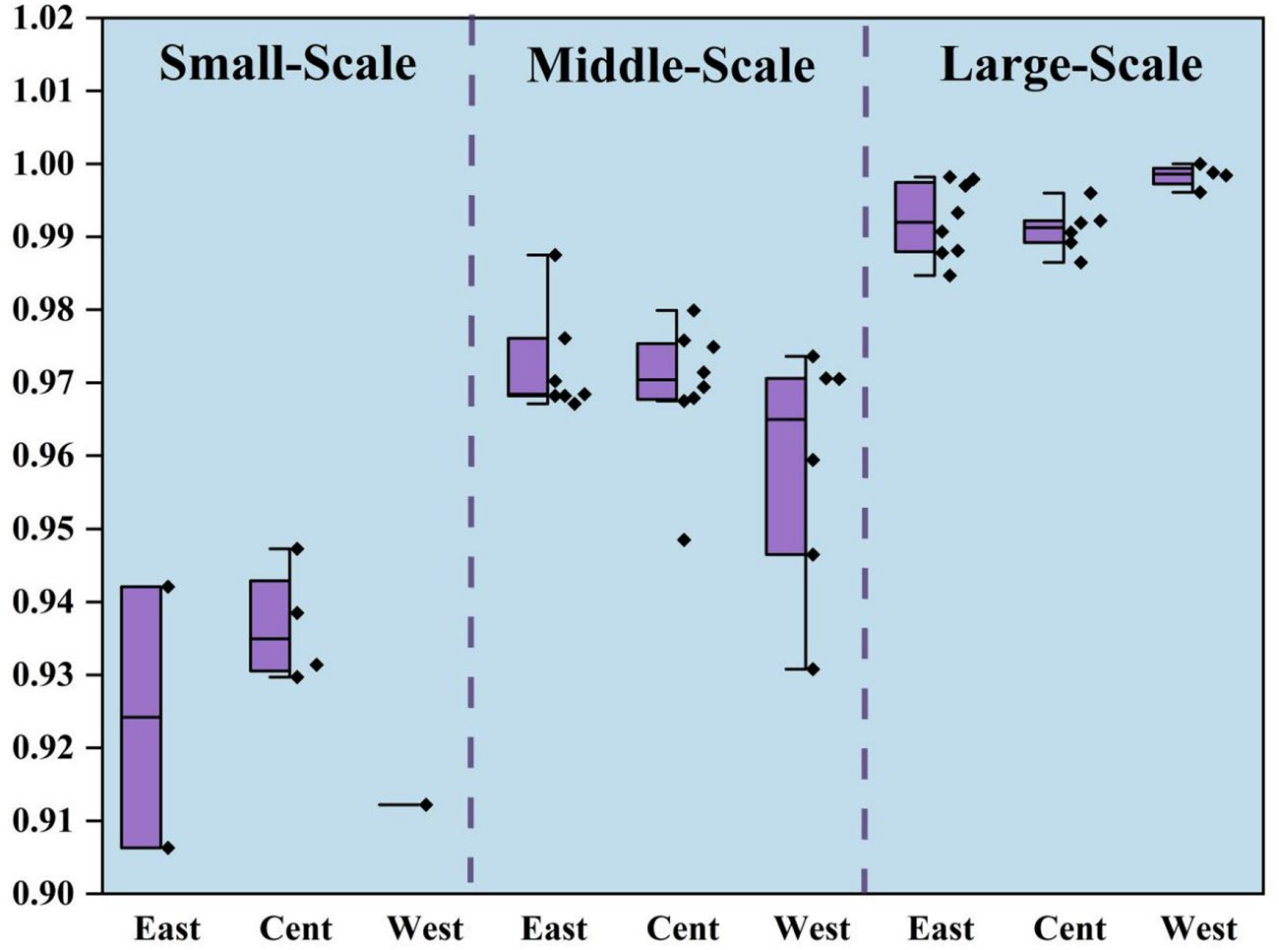
Three-scaled TGR in distinctive regions.

As shown in Fig. 7, the average TGR of small-scale eastern, central and western regions are 0.9242, 0.9367, and 0.9122 respectively, the average TGR of medium-sized eastern, central and western regions are 0.9723, 0.9686 and 0.9586 respectively, and the average TGR of large-scale eastern, central and western regions are 0.9922, 0.9911 and 0.9983 respectively. The average TGR of large-scale laying hens was closest to 1. From the regional point of view, the TGR in the western region is low (0.9564), which indicates that there is a big gap between the farming technology and the optimal low-carbon environmental protection breeding technology. This may be due to a series of reasons, such as extensive water use, low technical level, unreasonable breeding structure and so on. Along with the technological progress in the production process of laying hens, such as the changes of laying hens’ varieties and feeding techniques, these technological changes have improved the per unit area yield of laying hens. However, due to the differences in technological progress in different regions, technological progress will have different impacts on the egg production layout in various regions, resulting in different TGR. For a long time, the scattered breeding and the lack of industry norms have led to a certain degree of overcapacity in the laying hens feeding industry, thus resulting in the waste of resources and the lower price trend of egg prices for a long time. Traditional small-scale farmers are gradually divided into two levels, either exiting the market, or developing standardized scale farming by improving the production environment and updating machinery and equipment to reduce costs and expand production benefits to ensure corresponding profits. As the main production area of China’s laying hens, the large-scale breeding of laying hens in western provinces is in the stage of rapid development, and the water pollution load is large, however, the fact is that the level of fecal sewage treatment technology and heating technology lags behind. Therefore, it is necessary to have a certain resource tilt in this region, and focus on strengthening the input and efficiency level of production factors of laying hens breeding in the region.

## Conclusions and policy suggestions

Based on the MinDW-MML model, this paper constructs MML index by considering negative output and calculates China’s LHG from 2004 to 2018. The conclusion is as follows:

1. From 2004 to 2018, China’s middle-scale LHG was the highest, followed by small-scale and lowest in large-scale^35^. Although large-scale LHG is the lowest at present, it has the advantage of scale effect and great development potential in the future. In terms of regional distribution, the western region is the highest, followed by the central region, and the eastern region is the lowest. It indicates that the unique geographical conditions are also very important for the development of laying hens breeding industry, therefore it is crucial to make full use of the natural conditions.
2. From 2004 to 2018, China’s overall LHG showed a positive growth, and the decomposition indicators suggest efficiency decline and technological progress. The growth of small-scale LHG depends more on the improvement of efficiency, while the growth of medium-scale and large-scale LHG depends more on the technology progress. As a whole, the laying hens breeding industry is relatively fragile and is greatly affected by emergencies. In the first half of 2017, the outbreak of H7N9 caused a serious blow to the China’s laying hens feeding industry.
3. The results obtained under common frontier and group frontier are different. The LHG under common frontier is lower than the LHG under group frontier. Small-scale and middle-sized LHGs are greater than 1 under the group frontier. Under the common frontier, only middle-scale LHG is greater than 1, and LHG is obviously overestimated in group frontier. The TGR of large-scale laying hens breeding is the closest to 1, followed by small-scale and middle-scale, indicating that large-scale laying hens farming is the closest to the potential optimal technical environment efficiency level.

Based on the above empirical results, this paper proposes the following policy suggestions:

1. Attach importance to the development of middle-scale and determine the optimal scale of laying hens breeding according to local conditions. The middle-scale LHG is the highest, and the government should provide help and support in farming subsidies, financial credit and other preferential policies. However, due to the different economic conditions in China’s different regions and the different scale of laying hens breeding, it is impossible to establish a unified optimal scale of breeding efficiency in the whole country. This has no practical significance. Therefore, the government should determine the optimal scale of laying hens breeding efficiency based on the local actual situation, so as to promote the laying hens breeding according to local conditions.
2. In the process of laying hens raising, the efficiency level should be improved continuously. On the one hand, it is important to improve the utilization rate of existing technology and management level. The government should pay more attention to environmental protection and pollution emissions, while paying attention to economic development, and increase investment in science and technology and talent introduction, and constantly improve breeding technology. On the other hand, it needs to carry on scientific and reasonable breeding of laying hens. According to the diverse development and environmental conditions in different regions, it needs to select various kinds of laying hens for breeding, reasonably allocate feed, scientifically raise and manage, and fully improve the yield of laying hens.
3. Pay attention to the related work of epidemic prevention. The outbreak of H7N9 epidemic has caused a heavy blow to China’s laying hens breeding industry. Therefore, it is necessary to improve the relevant epidemic prevention policies, strengthen the awareness of epidemic prevention of breeding personnel, and make them understand the relevant epidemic prevention knowledge. In addition, the government should constantly enhance the publicity and education of epidemic prevention knowledge, so that breeding personnel can understand the importance and harm of epidemic prevention. It can effectively improve the survival rate of laying hens by avoiding the slackness of breeding personnel on epidemic prevention.

## Data Availability

The datasets generated and analysed during the current study are available in the “National Agricultural Product Cost and Benefit Data Compilation” and “Discharge Coefficient Manual” released by the Office of the First National Pollution Source Census Leading Group. https://data.cnki.net/yearbook/Single/N2019120280, https://wenku.baidu.com/view/9f82b6740342a8956bec0975f46527d3250ca66c.html.
